# Elevated expression of AGGF1 predicts poor prognosis and promotes the metastasis of colorectal cancer

**DOI:** 10.1186/s12885-019-6474-7

**Published:** 2019-12-27

**Authors:** Xin Zhang, Huimin Sun, Wanyuan Chen, Xianglei He

**Affiliations:** 1 Department of pathology, Zhejiang Provincial Peoples’ Hospital, Peoples’ Hospital of Hangzhou Medical College, Hangzhou, 310014 China; 2 Department of pathology, Weifang People’s Hospital, Weifang, 261041 China

**Keywords:** Colorectal cancer, AGGF1, Prognosis, Metastasis

## Abstract

**Background:**

Angiogenic factor with G-patch and FHA domains 1 (AGGF1) can promote angiogenesis and increasing evidence has highlighted the important roles of AGGF1 in tumorigenesis. However, the differential expression as well as the biological functions of AGGF1 in colorectal cancer (CRC) remain to be established. The purpose of the present study is therefore to identify the effect of AGGF1 on prognosis and metastasis in CRC patients.

**Methods:**

The expression level of AGGF1 in CRC was examined by qPCR, western blot and immunohistochemistry in a tissue microarray containing 236 CRC specimens and paired normal mucosae. And the effect of AGGF1 on CRC cell malignance was investigated in our established stable AGGF1 upregulated and knockdown CRC cell lines.

**Results:**

The expression level of AGGF1 in CRC tissue was not significantly different to that in adjacent normal mucosa at the mRNA level. However, at the protein level, AGGF1 expression in CRC tissues was significantly higher than in paired normal mucosa, which showed a clear association with TNM stage, AJCC stage, vascular invasion, and differentiation. Further, we revealed an apparent correlation between AGGF1 expression and poorer disease-free survival and overall survival of CRC patients. In addition, we discovered that AGGF1 significantly promoted CRC cell wound healing, migration, and invasion in vitro and distant metastasis in vivo.

**Conclusions:**

Our study demonstrates the aberrant overexpression of AGGF1 in CRC and provides a basis on which to explore the application of AGGF1 as a potential therapeutic target for CRC patients, especially for CRC patients with distant metastasis.

## Background

Colorectal cancer (CRC) is the third most commonly diagnosed cancer in males and the second in females around the world [1]. Attributed to CRC screening and/or a variety of therapies for advanced metastatic CRC being available, the mortality rates of CRC have decreased in recent years [1, 2]. However, outcomes remain suboptimal due to a lack of efficient targets for postponing, even preventing the invasion and metastasis of CRC [3]. Accordingly, discovering targets involved in CRC progression and elucidating the mechanism by which they induce metastasis appears to be particularly urgent.

Over the past decade, our understanding of vascular endothelial growth factor (VEGF), which is essential for both physiological and pathological angiogenesis, has increased at an explosive rate, leading to the approval of anti-angiogenic drugs for cancer [4, 5]. The angiogenic factor with G-patch and FHA domains 1 (AGGF1) protein is released outside endothelial cells when angiogenesis starts, promoting angiogenesis as strongly as VEGF [6, 7]. The full-length AGGF1 complementary DNA (4049 base pairs) contains a long open reading frame and encodes a protein of 714 amino acids [8, 9]. AGGF1 ubiquitously expresses in human normal cells as well as cancer cells [6]. Numerous studies have reported the aberrant expression of AGGF1 in cancer tissues and its involvement in malignant progression, such as in gastric cancer, hepatocellular carcinoma, and serous ovarian cancer [10–12]. However, whether AGGF1 also promotes the progression of CRC and the underlying role of AGGF1 in CRC progression remains to be elucidated.

In the present study, we first identified the expression patterns of AGGF1 at both the mRNA and the protein level in CRC tissues. Then, by examining AGGF1 expression in a tissue microarray, we evaluated the association between its expression and clinicopathologic features of CRC patients. Further cell functional assays in vitro and in vivo were used to explore the roles of AGGF1 in CRC cell migration and invasion, aiming to reveal the promoting roles of AGGF1 in the development of CRC, especially in patients with distant metastasis.

## Methods

### Patients and tissue specimens

A total of 236 CRC patient samples were included at the time of diagnosis from patients treated at departments of general surgery, Zhejiang Provincial People’s Hospital. Informed consent was obtained from all individual participants included in the study and the research was carried out in accordance with the World Medical Association Declaration of Helsinki. All patient-derived specimens were collected and archived under protocols approved by the institutional review boards of Zhejiang Provincial People’s Hospital. Procedures were carried out in accordance with approved guidelines. Cancer tissues and their paired adjacent normal mucosa were collected immediately after surgical resection, then frozen in liquid nitrogen for subsequent RNA extraction or formalin-fixed and paraffin-embedded for immunohistochemical staining.

### Quantitative PCR (qPCR)

The qPCR assays were performed as described previously [13–15]. The primers used for qPCR were: AGGF1, sense 5′-GGA CTA GTT TGC GAA AAC ATG GGT AGT G-3′ and antisense 5′-CCC AAG CTT TGC CTG CAA TGG TCT TTA TC-3′; GAPDH, sense 5′-GGG AAG GTG AAG GTC GGA GT-3′ and antisense 5′-GGG GTC ATT GAT GCRC AAC A-3′.

### Western blot (WB) analysis

The WB assays were performed as described previously [13–15]. Primary antibodies specific to AGGF1 (1:500, # ab203680) and GAPDH (1:1000, # ab8245) were purchased from Abcam (Cambridge, UK).

### Tissue microarray (TMA) construction and immunohistochemical (IHC) staining

TMA construction was undertaken as reported previously [15]. The expression of AGGF1 in the TMA was tested using standard IHC staining methods. The staining intensity for AGGF1 was scored as follows: 0 (negative), 1 (weak), 2 (moderate), 3 (strong); and staining extent, based on the percentage of positively stained cells, was scored as follows: 0 (0%), 1 (1–25%), 2 (26–50%), 3 (51–75%), 4 (76–100%). The sum of intensity and extent score were used as the final staining score: 0–2, negative expression; 3–4, weak expression; and 5–7, strong expression [16, 17]. The corresponding primary antibodies were: AGGF1 (1:100, Abcam, Cambridge, UK).

### Cell culture and transfection

Human CRC cell lines (SW480, SW620, HT-29, RKO, HCT-8, HCT-116, and LoVo) and a normal colon epithelial cell line (FHC) were purchased from the American Type Culture Collection (Manassas, VA, USA) in October 2018. All cell lines were cultured in Dulbecco’s modified Eagle’s medium (Gibco BRL, Grand Island, USA) supplemented with 10% fetal bovine serum, under a humidified atmosphere containing 5% CO_2_ at 37 °C. Cell morphology was authenticated via microscope (Fluorescence Microscope Axiovert 200, Zeiss, Germany).

For gene expression manipulation studies, both small interfering RNA (siRNA) specially targeting AGGF1 and pcDNA3.1-AGGF1 plasmid and their control sequences were obtained from Obio Technology Co., Ltd. (Shanghai, China). CRC cells were transfected using Lipofectamine 2000 following the manufacturer’s instructions.

### Colony formation assays

Colony formation assays were used to evaluate the colony formation abilities of CRC cells and colony formation was determined by preparing single cell suspension solutions and seeding in 6-well plate with 800 cells/well. After a 14-day incubation, the cell colonies were fixed in methyl alcohol for 15 min and dyed with crystal violet for 15 min. Colonies were then counted, and the plates were photographed.

### Wound healing assays

Transfected cells were trypsinized and seeded into 6-well plates (1.0 × 10^5^ cells/well). Upon reaching the exponential growth phase, cells were wounded with a sterile pipette tip and then washed with PBS. Images were captured at 0, 12, 24, and 36 h intervals, and wound widths were quantified and compared with baseline values. Experiments were carried out in triplicate.

### Transwell assays

The transwell 24-well Boyden chamber (Corning, USA) with an 8.0 μm pore size polycarbonate membrane was used for the cell migration (without Matrigel) and invasion (with Matrigel) assays according to the manufacturer’s protocol. Briefly, each group of cells (5 × 10^4^/chamber) was plated in the upper chambers in 200 μl serum-free media for 36 h, while the bottom chambers contained 600 μl media supplemented with 10% FBS as a chemoattractant. Cells that migrated and invaded to the reverse side of chamber inserts were fixed by methyl alcohol and stained with 0.1% crystal violet.

### Animal assays

To further investigate the metastatic effect of AGGF1 in vivo, we utilized a CRC metastasis model in four-week-old male, specific pathogen-free BALB/C nude mice, which were purchased from Shanghai Research Center for Model Organisms and housed under pathogen-free conditions in the animal experiment center of Zhejiang Provincial People’s Hospital. A total of 1 × 10^6^ (200 μl) HCT-116/Lv-AGGF1 and RKO/si-AGGF1 as well as their control cells were transfected with lenti-LUC virus and injected into the tail veins of grouped nude mice (*n* = 5), respectively. At weekly intervals, after ether inhalation, mice were subjected to i.p. injection with D-luciferin (150 mg/kg), then imaged 10 min later using the IVIS Illumina System (Caliper Life Sciences). After six weeks, the mice were euthanized by cervical dislocation. The lung and liver tissue samples were obtained, weighed, and embedded in paraffin. The in vivo assay using nude mice was approved by the Institutional Animal Care and Use Committee of Zhejiang Provincial People’s Hospital. All animal protocols were approved by Zhejiang Provincial People’s Hospital Animal Care.

### Statistical analysis

The Student’s t-test was used to determine the differences in AGGF1 mRNA expression between CRC tissues and adjacent normal mucosa. The statistical significance of differences between AGGF1 expression and clinicopathological variables was estimated by the χ^2^ test or Fisher’s exact test. Survival curves were calculated by the Kaplan–Meier method with the log-rank test. The hazard ratio with a 95% confidence interval in Cox proportional hazards regressions were applied to estimate the hazard risk of individual factors for overall survival (OS) and disease-free survival (DFS). *p* < 0.05 with a two-sided test was considered to be statistically significant. All statistical analyses were carried out using the SPSS 22.0 statistical software package (SPSS, Chicago, IL, USA).

## Results

### AGGF1 protein rather than mRNA showed aberrant expression in CRC

**Fig. 1:**
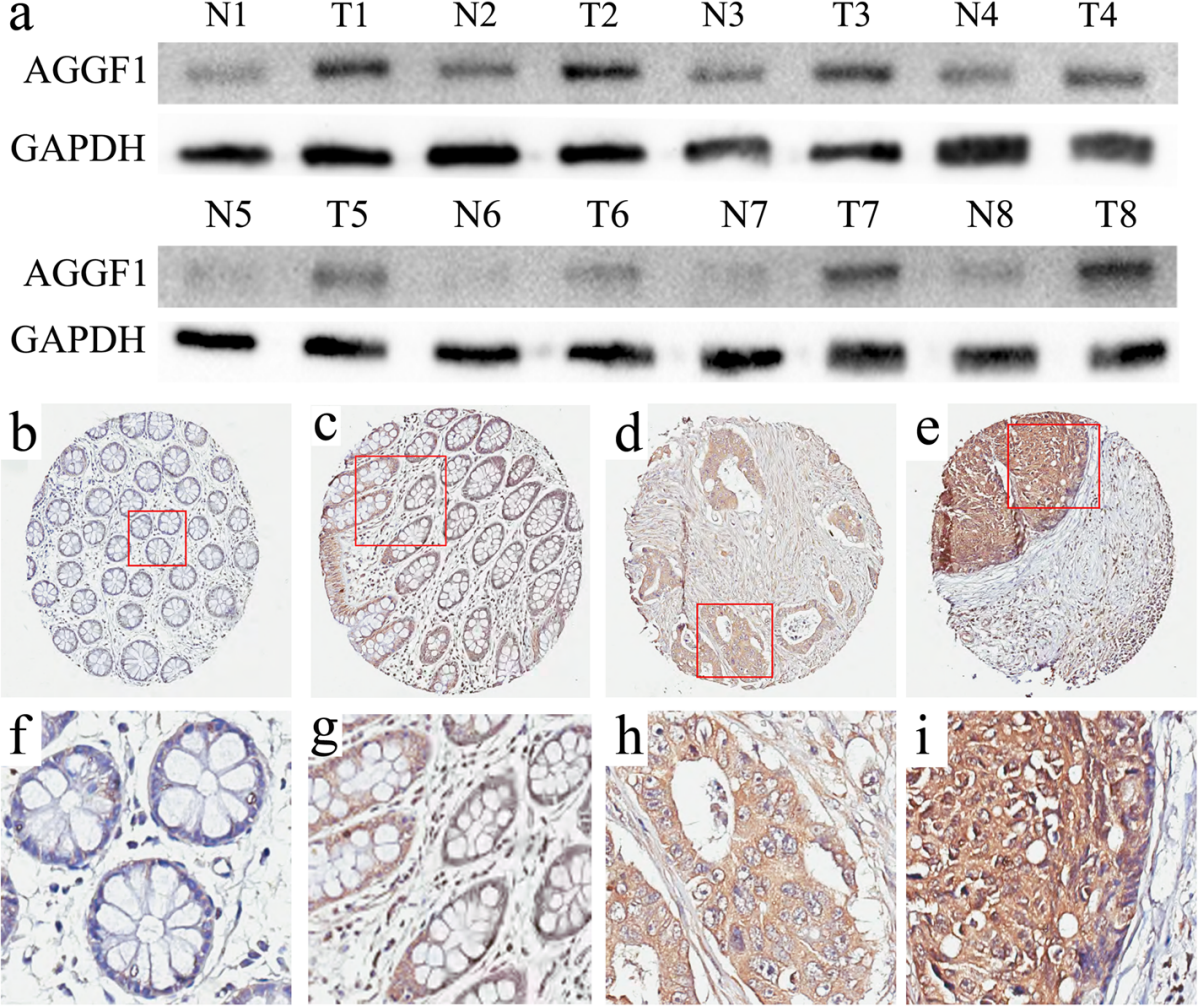
Expression of AGGF1 in CRC tissues and paired normal mucosa. **a**. Western blot analysis was used to detect AGGF1 protein expression in eight representative paired CRC tissue samples, with GAPDH used as the loading control. **b–i:** Immunohistochemical staining showed that AGGF1 protein expression was significantly higher in CRC tissues compared with adjacent normal mucosa, with AGGF1 staining mainly observed in the cytoplasm of CRC cells. **b, f:** Negative AGGF1 staining in normal colorectal epithelium; **c, g:** Weak AGGF1 staining in well-differentiated CRC; **d, h:** Intense AGGF1 staining in moderately differentiated CRC; **e, i:** Strong AGGF1 staining in poorly differentiated CRC; **b–e.** Original magnification: 50×; **f–i.** Original magnification: 400 ×

Using qPCR, we first detected the expression level of AGGF1 in 30 pairs of randomly selected CRC specimens. However, no clear difference was observed between CRC tissues and paired normal mucosa at the mRNA level (Additional file 1: Fig. S1). Consistent with this, upon further analysis of four public databases from Oncomine (https://www.oncomine.org/resource/main.html) we did not find significantly different AGGF1 mRNA expression between CRC tissues and normal mucosa (Additional file 2: Fig. S2). Subsequently, we examined the protein level of AGGF1 in eight samples randomly selected from the 30 paired specimens mentioned above by WB assay and found that the expression of AGGF1 protein was significantly upregulated in CRC tissues compared to paired normal mucosa (Fig. 1a). Taken together, these data suggest that aberrant expression of AGGF1 in CRC tumorigenesis and development may play significant roles at the protein level rather than at the mRNA level.

### Correlations between AGGF1 protein expression and clinicopathological features in CRC

To clarify the correlations between AGGF1 protein expression and clinicopathological features of CRC patients, we first examined the expression of AGGF1 in a TMA by immunohistochemical (IHC) staining. As summarized in Additional file 6: Table S1, of the 236 normal specimens in the TMA, 129 (54.7%) showed negative AGGF1 expression (Fig. 1b,f and control experiments were showed in Additional file 3: Fig. S3), 68 (28.8%) had weak positive staining, and 39 (16.5%) displayed strong positive staining. In contrast, the immunoreactive patterns of AGGF1 were predominantly positive in the majority of CRC specimens, with 76 (32.2%) cases showing weak positive staining and 114 (48.3%) exhibiting strong positive staining (Fig. 1c-e and g-i). These results further confirm that the expression levels of AGGF1 in CRC tissues were higher than those in adjacent normal mucosa (*p* < 0.001).

The correlation between AGGF1 expression and clinicopathologic features of CRC patients is summarized in Table 1. Aberrant upregulated expression of AGGF1 was highly correlated with tumor invasion (*p* < 0.001), LNM (*p* < 0.001), distant metastasis (*p* = 0.012), advanced AJCC stage (*p* < 0.001), and tumor recurrence (*p* = 0.004). On the other hand, no significant association was found between AGGF1 expression and age, gender, tumor location, or differentiation (*p* > 0.05 for all, Table 1). All of these data further reveal the aberrant expression of AGGF1 in CRC tissues and its association with a more malignant CRC phenotype.

**Table 1:** Associations between AGGF1 expression and clinicopathological characteristics in 236 CRC patients

Variable	n	AGGF1 expression			*p* value
		Negative (46)	Weak positive (76)	Strong positive (114)	
Age (yr)					0.837
< 57	112	22 (19.6%)	34 (30.4%)	56 (50.0%)	
> =57	124	24 (19.4%)	42 (33.9%)	58 (46.8%)	
Gender					0.359
Male	139	23 (16.5%)	45 (32.4%)	71 (51.1%)	
Female	97	23 (23.7%)	31 (32.0%)	43 (44.3%)	
Tumor location					0.381
Right	49	5 (10.2%)	15 (30.6%)	29 (59.2%)	
Transverse	5	1 (20.0%)	2 (40.0%)	2 (40.0%)	
Left	12	2 (16.7%)	3 (25.0%)	7 (58.3%)	
Sigmoid	49	7 (14.3%)	16 (32.7%)	26 (53.1%)	
Rectum	121	31 (25.6%)	40 (33.1%)	50 (41.3%)	
T classification					< 0.001*
T 1	2	2 (100.0%)	0 (0.0%)	0 (0.0%)	
T 2	36	21 (58.3%)	7 (19.4%)	8 (22.2%)	
T 3	44	7 (15.9%)	21 (47.7%)	16 (36.4%)	
T 4	154	16 (10.4%)	48 (31.2%)	90 (58.4%)	
N classification					< 0.001*
N 0	108	36 (33.3%)	44 (40.7%)	28 (25.9%)	
N 1	77	7 (9.1%)	18 (23.4%)	52 (67.5%)	
N 2	51	3 (5.9%)	14 (27.5%)	34 (66.7%)	
M classification					0.012*
M 0	204	44 (21.6%)	69 (33.8%)	91 (44.6%)	
M 1	32	2 (6.3%)	7 (21.9%)	23 (71.9%)	
AJCC stage					< 0.001*
I	24	19 (79.2%)	5 (20.8%)	0 (0.0%)	
II	76	15 (19.7%)	36 (47.4%)	25 (32.9%)	
III	104	10 (9.6%)	28 (26.9%)	66 (63.5%)	
IV	32	2 (6.3%)	7 (21.9%)	23 (71.9%)	
Recurrence					0.004*
No	199	43 (21.6%)	69 (34.7%)	87 (43.7%)	
Yes	37	3 (8.1%)	7 (18.9%)	27 (73.0%)	
Differentiation					0.640
Well	50	8 (16.0%)	17 (34.0%)	25 (50.0%)	
Moderate	173	34 (19.7%)	57 (32.92%)	82 (47.4%)	
Poor	13	4 (30.8%)	2 (15.4%)	7 (53.8%)	

*Significant difference

### High expression of AGGF1 predicted poorer prognosis in CRC

**Fig. 2:**
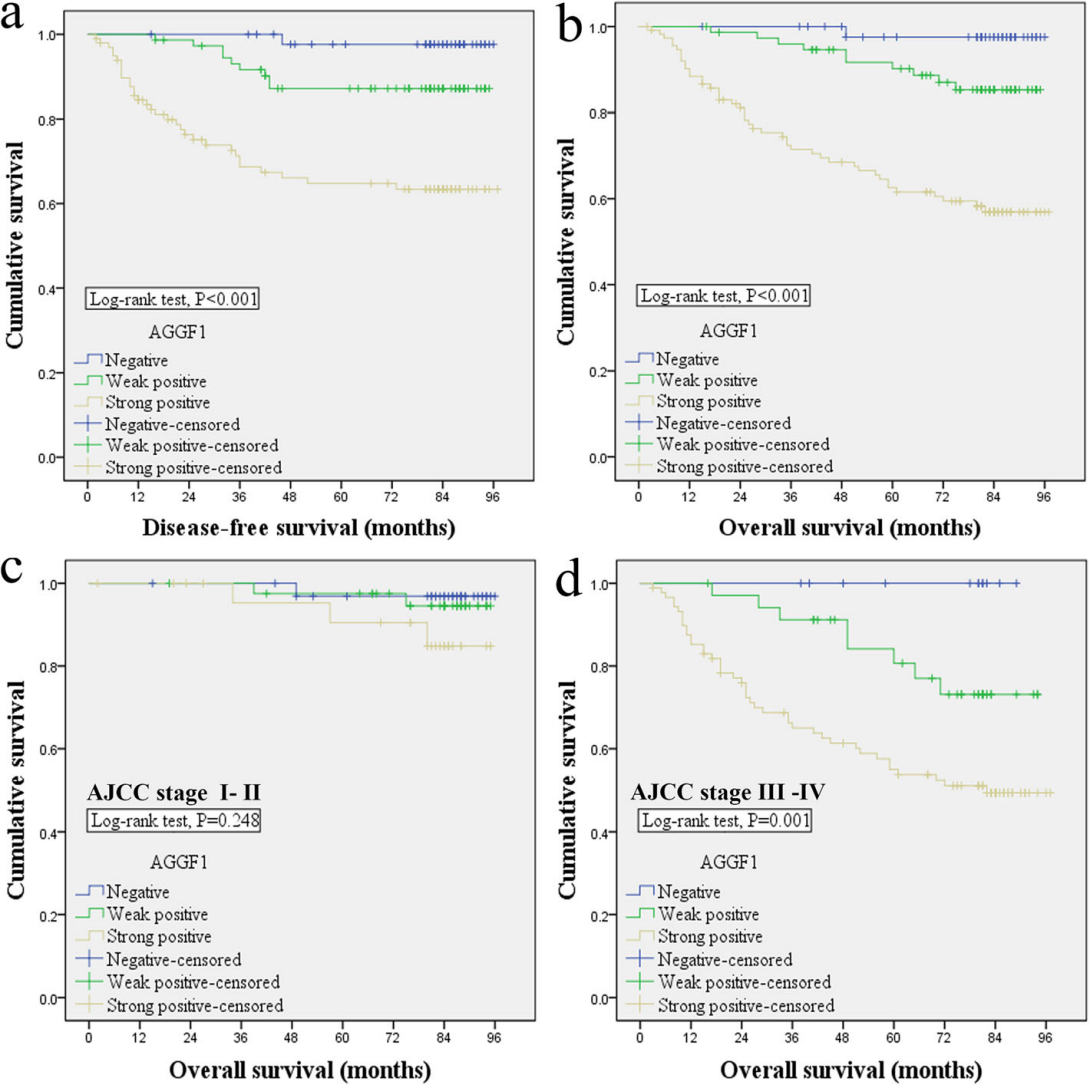
Kaplan–Meier analysis with a log-rank test of survival. Disease-free survival (**a**) and overall survival (**b**) were significantly shorter in patients with AGGF1-positive tumors than in those with AGGF1-negative tumors (*p < 0.05 for both, log-rank test). *P*-values were calculated by log-rank test and *p* < 0.05 was considered significant

Using Kaplan–Meier curves with a log-rank test for disease-free survival (DFS) and overall survival (OS), we assessed the predictive role of AGGF1 in CRC patient survival and found that patients with AGGF1-positive tumors had poorer DFS and OS rates than those with AGGF1-negative tumors (*p* < 0.05 for both, Fig. 2a-b). More interestingly, in CRC patients at AJCC stage III-IV, higher AGGF1 expression was significantly associated with poorer OS (*p* = 0.001, Fig. 2d), while no significant difference between higher and lower AGGF1 expression was observed for OS in patients at AJCC stage I- II, (*p* = 0.2248, Fig. 2c). Additionally, univariate and multivariate survival analyses for DFS and OS were performed using the Cox proportional hazards model and the results demonstrated that AGGF1 expression (*p* < 0.05, Table 2 and Table 3) could serve as an independent prognostic factor for DFS and OS in CRC patients. Interestingly, we also analyzed the impact of aberrant AGGF1 mRNA expression on CRC patient survival by using the public dataset from TCGA and discovered that there was no significant association between the expression of AGGF1 and OS (Additional file 4: Fig. S4), which further demonstrates that AGGF1 plays significant roles in tumorigenesis and the progression of CRC at the protein level rather than the mRNA level.

**Table 2:** Univariate and multivariate analysis of disease-free survival in 236 CRC patients

	Disease-free survival			
	Univariate analysis		Multivariate analysis	
	HR (95% CI)	*p* Value	HR (95% CI)	*p* Value
Age (yr)	0.871 (0.476–1.595)	0.655		
Gender	1.153 (0.628–2.117)	0.646		
Tumor location	0.997 (0.816–1.220)	0.980		
T classification	2.636 (1.415–4.911)	0.002*	2.773 (1.238–6.211)	0.013*
N classification	2.514 (1.480–3.136)	< 0.001*	0.744 (0.415–1.334)	0.007*
M classification	6.733 (3.281–13.815)	< 0.001*	9.195 (3.931–21.504)	< 0.001*
AJCC stage (III -IV vs I- II)	6.160 (2.593–14.635)	< 0.001*	3.968 (1.203–130.90)	0.024*
Recurrence	7.689 (4.112–14.375)	< 0.001*	7.422 (3.701–14.884)	< 0.001*
Differentiation	0.666 (0.368–1.206)	0.180		
AGGF1	3.956 (2.179–7.182)	< 0.001*	3.268 (1.779–6.002)	< 0.001*

HR: hazard ratio; CI: confidence interval

* Significant differences

**Table 3:** Univariate and multivariate analysis of overall survival in 236 CRC patients

	Overall survival			
	Univariate analysis		Multivariate analysis	
	HR (95% CI)	*p* Value	HR (95% CI)	*p* Value
Age (yr)	0.895 (0.530–1.512)	0.679		
Gender	1.222 (0.723–2.066)	0.455		
Tumor location	1.168 (1.000–1.364)	0.050*	1.190 (0.994–1.423)	0.058
T classification	3.182 (1.705–5.937)	< 0.001*	2.938 (1.404–6.149)	0.004*
N classification	2.243 (1.615–3.115)	< 0.001*	0.912 (0.586–1.419)	0.002*
M classification	9.412 (5.481–16.162)	< 0.001*	9.385 (4.907–17.952)	< 0.001*
AJCC stage (III -IV vs I- II)	7.927 (3.394–18.511)	< 0.001*	3.369 (1.147–9.895)	0.027*
Recurrence	3.730 (2.144–6.488)	< 0.001*	5.667 (2.882–11.140)	< 0.001*
Differentiation	0.616 (0.370–1.025)	0.062		
AGGF1	4.182 (2.384–7.333)	< 0.001*	3.320 (1.814–6.077)	< 0.001*

HR: hazard ratio; CI: confidence interval

* Significant differences

### AGGF1 promotes CRC cell migration and invasion in vitro and distant metastasis in vivo

**Fig. 3:**
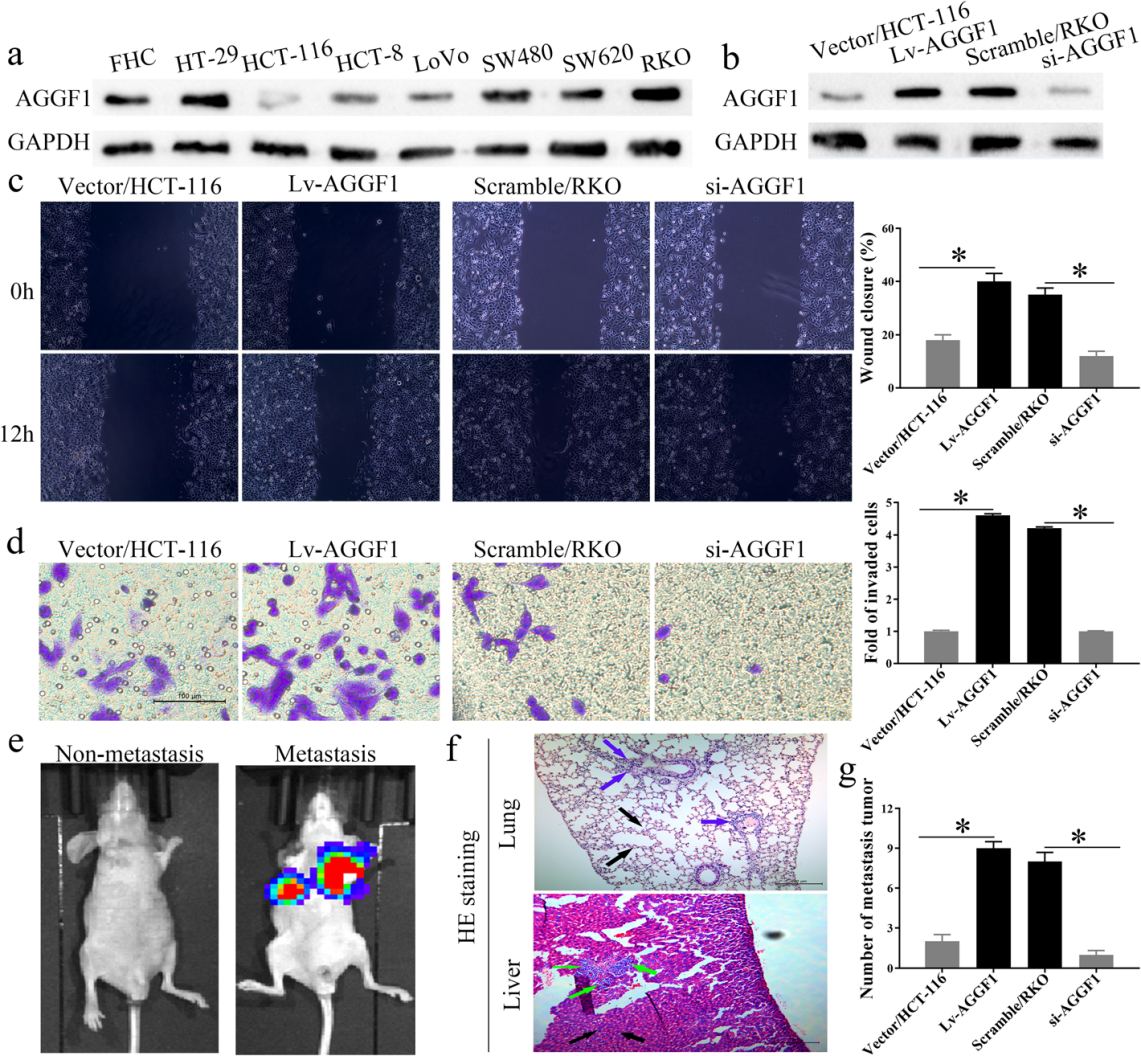
In vitro and in vivo assays. **a.** he protein level of AGGF1 in one normal colorectal mucosa cell line (FHC) and seven CRC cell lines. The expression level of AGGF1 protein in HCT-116 and RKO cells transfected with AGGF1 overexpression or knockdown vectors was validated using western blotting (**b**). GAPDH was used to normalize protein level. In vitro, overexpression or knockdown of AGGF1 inhibited or elevated CRC wound healing (**c**), migration, and invasion (**d**), respectively, compared with control groups. Upregulation or knockdown of AGGF1 expression facilitated or suppressed the tumor metastasis ability of CRC cells in nude mice. **e.** Representative images of metastatic tumor colonies in mice (left panel (non-metastasis): the nude mouse without metastatic tumor colonies formed, right panel (metastasis): the nude mouse with metastatic tumor colonies formed). **f.** HE staining of the metastatic tumor colonies in lung and liver (colored arrows: metastatic tumor colonies). **g.** The number of metastatic tumor colonies was counted (*p < 0.05, **c, f:** Original magnification: 100×; **d:** Original magnification: 200×)

To further investigate the roles of AGGF1 on CRC progression, we first detected the expression of AGGF1 in seven CRC cell lines and one healthy colorectal cell line at the protein level. Then, according to the discrepant expression levels of AGGF1, we selected HCT-116 and RKO, which showed the lowest and highest expression level of AGGF1, respectively, to further investigate the effect of AGGF1 on CRC biological processes (Fig. 3a). Subsequently, by viral transfection, we generated a stable AGGF1-overexpressing HCT-116 cell line and an AGGF1-knockdown RKO cell line, with the ideal efficiency evaluated by WB (Fig. 3b).

Firstly, we performed cell colony formation assays to explore the underlying roles of AGGF1 on CRC cells’ proliferation, and found that overexpression or knockdown of AGGF1 expression showed no significant promotion or inhibition on CRC cells’ proliferation, respectively (Additional file 5: Fig. S5). Then, in vitro wound healing assays showed that ectopic overexpression or knockdown of AGGF1 could significantly accelerate or delay the wound healing ability of CRC cells, respectively, as compared with the control groups (*p* < 0.05 for all, Fig. 3c). Transwell assays further demonstrated that upregulated or downregulated expression of AGGF1 could also strengthen or attenuate the migration and invasion ability, respectively, of CRC cells in vitro (*p* < 0.05 for all, Fig. 3d). To investigate the functional impact of AGGF1 on distant metastasis of CRC in vivo, in vivo fluorescence imaging of mice was performed to test the lung and liver metastasis of CRC (Fig. 3e). Then, hematoxylin-eosin (HE) staining was used to count the number of tumor colonies in mice lung and liver tissues (Fig. 3f). We found that AGGF1 overexpression or knockdown significantly aggrandized or weakened the formation of in vivo metastatic colonies compared with the control groups in both the lungs and livers of mice (Fig. 3g), *p* < 0.001). Taken together, these data indicate that AGGF1 could facilitate the wound healing, migration, and invasion abilities of CRC cell in vitro and also promote distant metastasis in vivo.

## Discussion

The prognosis for primary CRC is always favorable after curable surgical resection; however, the five-year OS rate of CRC patients with distant metastasis dramatically declines due to a lack of efficient drugs or treatment targets for postponing, even preventing the progression of CRC [18, 19]. Thus, the identification of novel targets for CRC and further elucidation the mechanisms involved in the distant metastasis appears to be vitally important. In the present study, we demonstrated that the expression of AGGF1 was elevated in CRC tissues compared with corresponding normal mucosae, but only at the protein level. Aberrant overexpression of AGGF1 predicted poorer DFS and OS in CRC patients after radical surgery. Further, we verified that AGGF1 could accelerate CRC cell wound healing, migration, and invasion in vitro as well as distant metastasis in vivo. All of these findings support the hypothesis that ectopic expression of AGGF1 promotes CRC metastasis, serving as a potential therapeutic target for CRC patients, especially for patients with lung and liver metastasis.

Located on human chromosome 5q13.3, the AGGF1 mRNA gene encodes a protein consisting of 714 amino acids that shows strong expression in blood vessels and is secreted as vessel formation initiates [6, 20]. A series of studies have also reported that the expression level of AGGF1 in cancer tissues was clearly higher than in adjacent normal tissues, predicting poor prognosis [10–12]. In the present study, at the mRNA level, we found no difference in the AGGF1 expression between the CRC tissues and paired normal tissues, a result which was consistent with public databases from Oncomine. However, at the protein level, the expression level of AGGF1 in CRC tissues/cells was much higher than in corresponding normal mucosa/cells. Further results from IHC staining in a TMA revealed that positive AGGF1 protein expression was significantly correlated with poorer DFS and OS in CRC patients. However, public data from TCGA showed no obvious correlation between aberrant AGGF1 expression and OS, suggesting that AGGF1 is vitally importance in CRC malignant progression at the protein level rather than at the mRNA level.

As reported, AGGF1 is released outside endothelial cells when angiogenesis starts, promoting angiogenesis [6, 7], and increased angiogenesis is involved in the growth, metastasis, and survival of various tumors [21, 22]. Reduced expression of AGGF1 resulted in endothelial cell apoptosis and inhibition of endothelial capillary vessel formation and cell migration, which could be rescued by purified recombinant human AGGF1 protein [7]. To further elucidate the role of AGGF1 in CRC progression, we built the CRC cell lines HCT-116 and RKO with AGGF1 stable overexpression and knockdown, respectively. In vitro, we determined that AGGF1 promoted CRC cell wound healing, migration, and invasion, which indicates the potential involvement of AGGF1 in CRC metastasis and is consistent with the results of studies in gastric cancer [10] and hepatocellular carcinoma [11]. Additionally, results from the in vivo assay in nude mice showed that up- or downregulation of AGGF1 expression also led to a significant increase or reduction, respectively, in metastatic colonies formed in lungs and livers compared with the control groups. These in vivo and vitro data suggest that the elevated expression of AGGF1 is likely correlated with tumor invasion.

Tumor is known as a multi-gene/multi-step process. There is no doubt about the importance of TNM-stage in tumor, however, even patients with the same TNM-stage may have different prognosis. Therefore, other molecules are still needed to supplement the TNM-stage to predict the prognosis and achieve accuracy study. For example, the immune scoring system can improve the assessment of the risk of CRC recurrence by introducing immune parameters for tumor staging [23]. The aberrant overexpression of AGGF1 in CRC can be detected by preoperative biopsy or postoperative immunohistochemistry, facilitating it served as a supplement to TNM staging. When patients with the same TNM-stage are accompanied by high expression of AGGF1, further intensive treatment should be specified to improve the prognosis of patients. Pre-operative radiotherapy or chemoradiotherapy is frequently used prior to CRC surgery to improve local control and survival [24]. The study on the clinical significance of AGGF1 in CRC patients with distant metastasis is conducive to the designation of personalized treatment strategy for CRC patients. Neo-adjuvant chemoradiotherapy could be adopted according to the patients’ personal conditions, so as to achieve the degraded treatment of CRC and improve the postoperative outcomes of CRC patients.

## Conclusions

We demonstrated that AGGF1 expression was aberrantly elevated in CRC tissues and showed significant correlations with poor DFS and OS in CRC patients. In addition, we clarified that AGGF1 could promote CRC cell wound healing, migration, and invasion in vitro and distant metastasis in vivo, which indicates that AGGF1 may function as a potential therapeutic target for CRC patients, especially for patients with distant metastasis. Future studies will focus on the mechanisms underlying the role of AGGF1 in the progression of CRC and the potential for targeting AGGF1 in CRC treatment.

## Supplementary information

**Additional file 1: Figure S1.** The mRNA level of AGGF1 expression was detected in 30 pairs CRC specimens by qPCR. GAPDH was used as an internal control (#*p*>0.05).

**Additional file 2: Figure S2.** Expression of AGGF1 at the mRNA level in four public databases from Oncomine. a. Ki Colon dataset, which is grouped as follows: no value (n=41), colon squamous cell carcinoma (n=3), colon adenocarcinoma (n=77), and gastrointestinal stromal tumor (n=2). b. Graudens Colon dataset, which is grouped as follows: no value (n=12) and colorectal carcinoma (n=48). c. Gaspar Colon dataset, which is grouped as follows: no value (n=22) and colorectal adenoma (n=56). d. Sabates-Bellver Colon dataset, which is grouped as follows: no value (n=32), rectal adenoma(n=7), and colon adenoma (n=25).

**Additional file 3: Figure S3.** Control experiments for antibody staining. a. Original magnification: 50×; b. Original magnification: 400×.

**Additional file 4: Figure S4.** Impact of AGGF1 expression at the RNA level on the OS of CRC patients in TCGA public databases. No significant correlation between aberrant expression of AGGF1 at the RNA level and OS was observed in CRC patients regardless of classification: a. Lower quartile classification; b. Lower tertile classification; c. Mean classification; d. Median classification; e. Upper quartile classification; f. Upper tertile classification.

**Additional file 5: Figure S5.** The impact of AGGF1 on the proliferation of CRC cells was detected by cell colony formation assays in vitro. The results showed the impact of overexpression (a) as well as knockdown (b) of AGGF1 on CRC cells' growth, with the number of CRC cell clones were quantified (c).

**Additional file 6: Table S1.** Expression of AGGF1 in normal colorectal mucosa and primary cancerous tissues (*n* = 236).

## Data Availability

The datasets used and analyzed during the current study are available from the corresponding author on reasonable request.

## Abbreviations

AGGF1: Angiogenic factor with G-patch and FHA domains 1
CRC: Colorectal cancer
DFS: Disease-free survival
IHC: Immunohistochemical
OS: Overall survival
TMA: Tissue microarray
VEGF: Vascular endothelial growth factor
WB: Western blot
