# Supplementary figures and images for: Elevated expression of AGGF1 predicts poor prognosis and promotes the metastasis of colorectal cancer
Source: BMC Cancer. 2019 Dec 27;19:1252. doi: 10.1186/s12885-019-6474-7 (PMC6935059; doi:10.1186/s12885-019-6474-7)

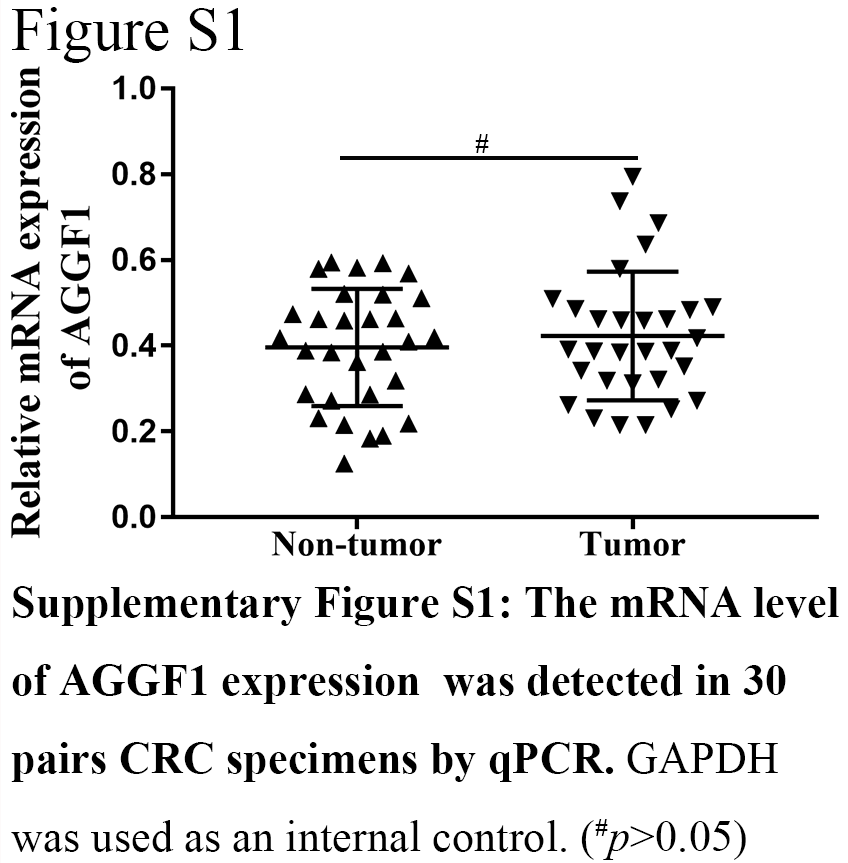

Supplement: Supplementary file 1 — Additional file 1: Figure S1. The mRNA level of AGGF1 expression was detected in 30 pairs CRC specimens by qPCR. GAPDH was used as an internal control (#p>0.05). [file 12885_2019_6474_MOESM1_ESM.tif]

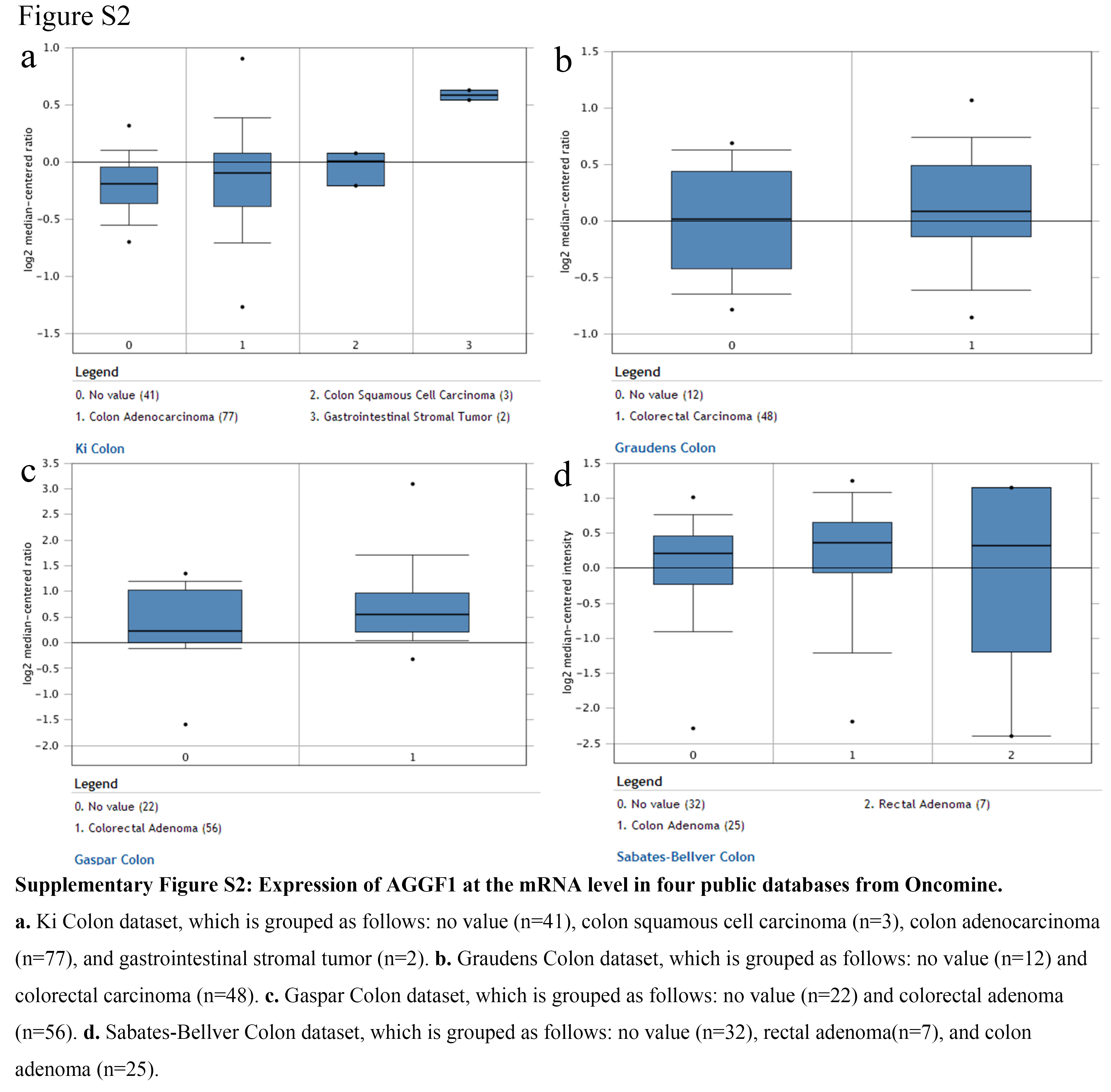

Supplement: Supplementary file 2 — Additional file 2: Figure S2. Expression of AGGF1 at the mRNA level in four public databases from Oncomine. a. Ki Colon dataset, which is grouped as follows: no value (n=41), colon squamous cell carcinoma (n=3), colon adenocarcinoma (n=77), and gastrointestinal stromal tumor (n=2). b. Graudens Colon dataset, which is grouped as follows: no value (n=12) and colorectal carcinoma (n=48). c. Gaspar Colon dataset, which is grouped as follows: no value (n=22) and colorectal adenoma (n=56). d. Sabates-Bellver Colon dataset, which is grouped as follows: no value (n=32), rectal adenoma(n=7), and colon adenoma (n=25). [file 12885_2019_6474_MOESM2_ESM.tif]

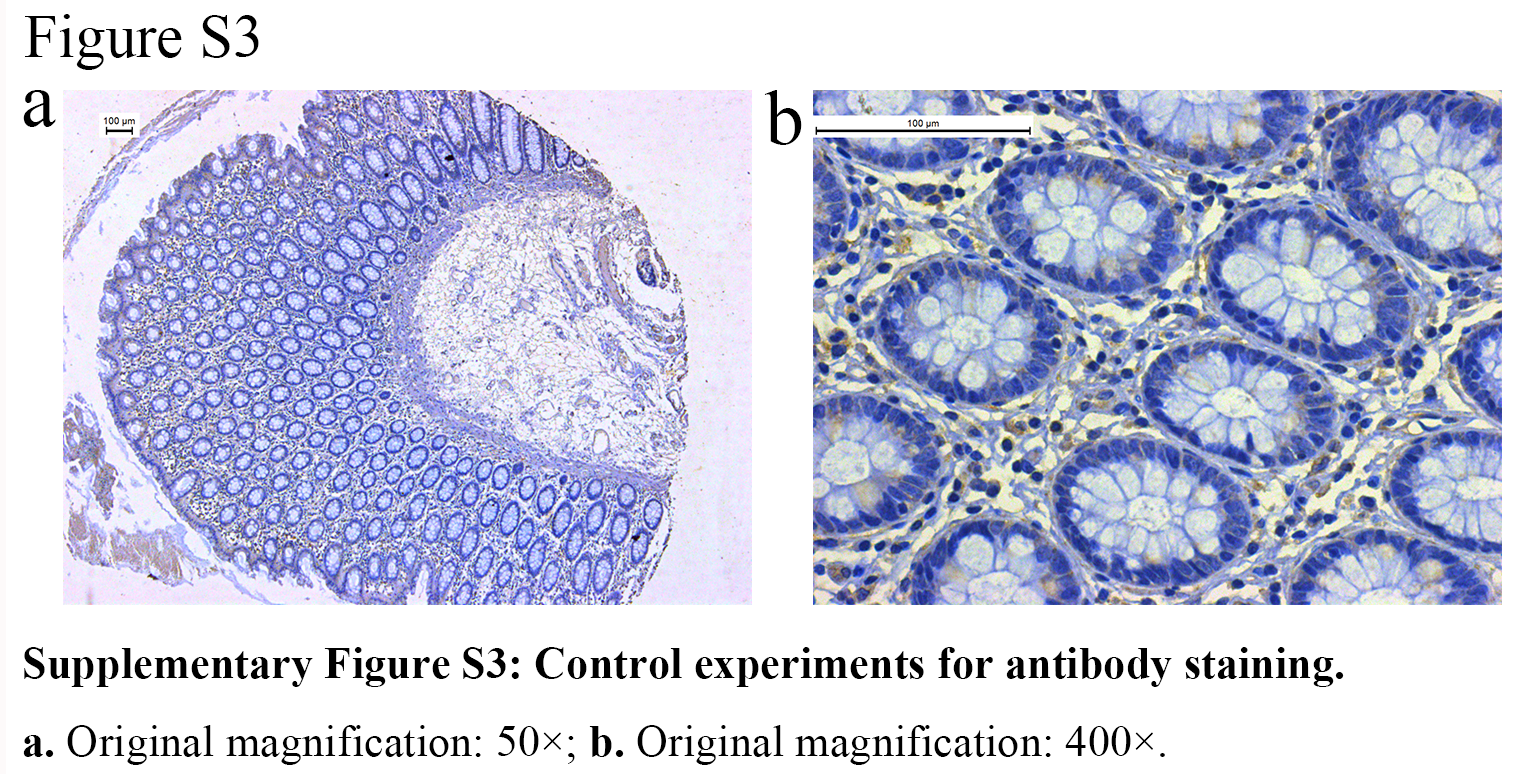

Supplement: Supplementary file 3 — Additional file 3: Figure S3. Control experiments for antibody staining. a. Original magnification: 50×; b. Original magnification: 400×. [file 12885_2019_6474_MOESM3_ESM.tif]

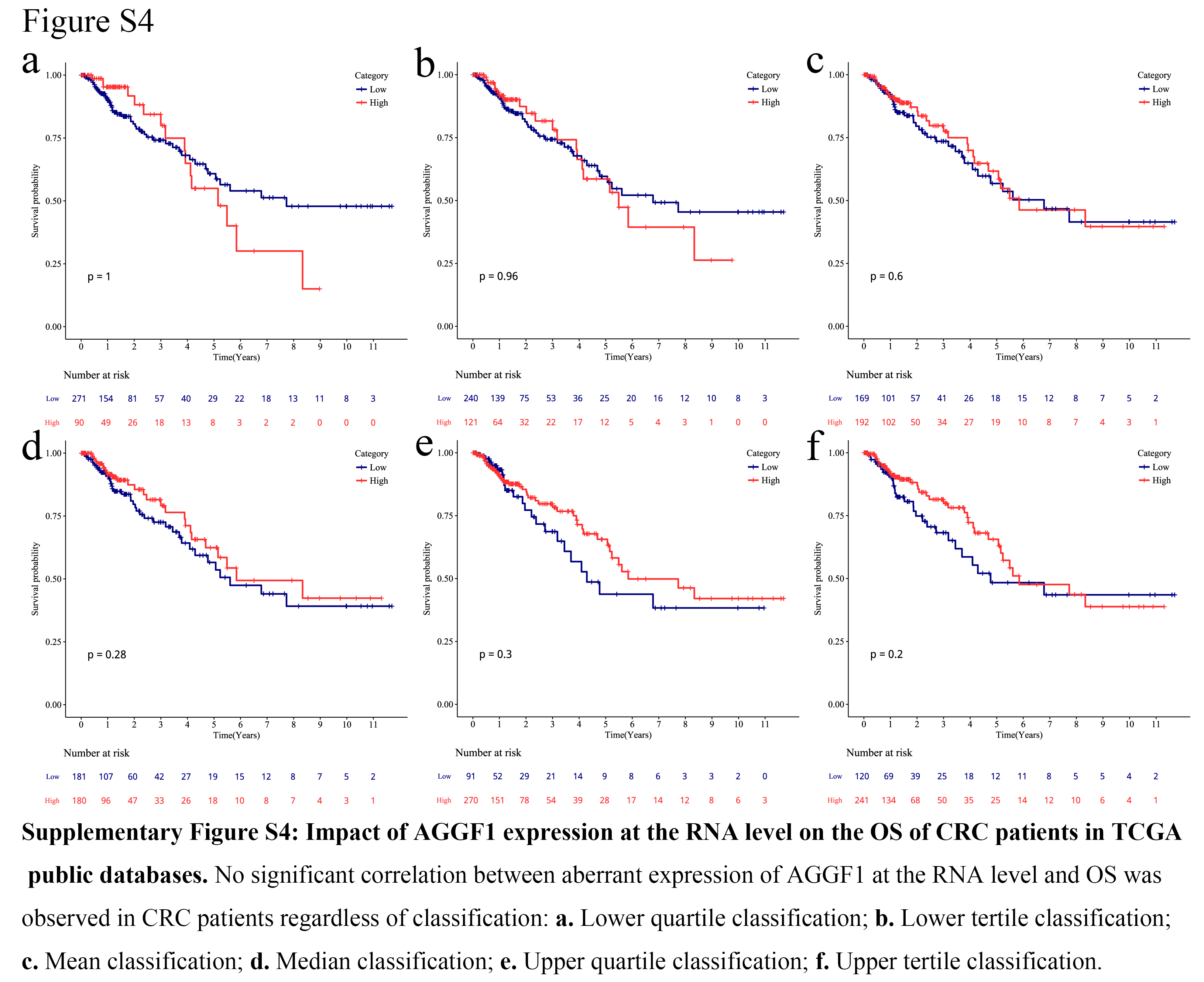

Supplement: Supplementary file 4 — Additional file 4: Figure S4. Impact of AGGF1 expression at the RNA level on the OS of CRC patients in TCGA public databases. No significant correlation between aberrant expression of AGGF1 at the RNA level and OS was observed in CRC patients regardless of classification: a. Lower quartile classification; b. Lower tertile classification; c. Mean classification; d. Median classification; e. Upper quartile classification; f. Upper tertile classification. [file 12885_2019_6474_MOESM4_ESM.tif]

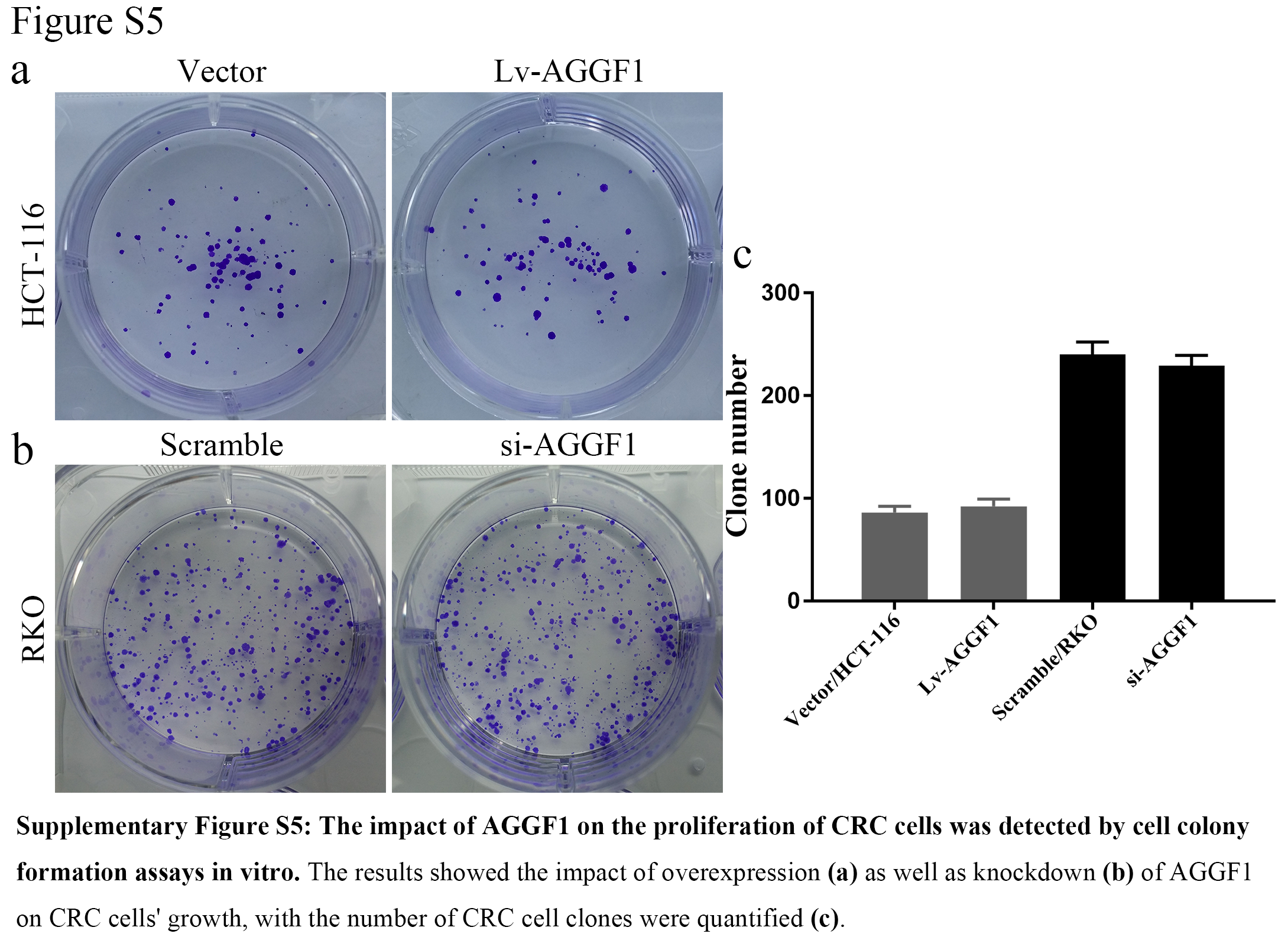

Supplement: Supplementary file 5 — Additional file 5: Figure S5. The impact of AGGF1 on the proliferation of CRC cells was detected by cell colony formation assays in vitro. The results showed the impact of overexpression (a) as well as knockdown (b) of AGGF1 on CRC cells' growth, with the number of CRC cell clones were quantified (c). [file 12885_2019_6474_MOESM5_ESM.tif]
