# Supplementary material for: Elevated expression of AGGF1 predicts poor prognosis and promotes the metastasis of colorectal cancer
Source: BMC Cancer. 2019 Dec 27;19:1252. doi: 10.1186/s12885-019-6474-7 (PMC6935059; doi:10.1186/s12885-019-6474-7)
Supplement: Supplementary file 6 — Additional file 6: Table S1. Expression of AGGF1 in normal colorectal mucosa and primary cancerous tissues (n = 236). [file 12885_2019_6474_MOESM6_ESM.docx]

**Supplementary Table S1.** Expression of AGGF1 in normal colorectal mucosa and primary cancerous tissues (n = 236).

| Tissue sample | n | Expression of AGGF1 | | | *p* value |
| --- | --- | --- | --- | --- | --- |
|  | | Negative (n, %) | Weak positive (n, %) | Strong positive (n, %) |  |
| Normal mucosa | 236 | 129 (54.7) | 68 (28.8) | 39 (16.5) | <0.001* |
| Tumor tissue | 236 | 46 (19.5) | 76 (32.2) | 114 (48.3) |  |

* Significant difference
